# The effect of vitamin C on mice hemolytic anemia induced by phenylhydrazine: an animal model study using histological changes in testis, pre-implantation embryo development, and biochemical changes

**DOI:** 10.22038/IJBMS.2018.25819.6356

**Published:** 2018-07

**Authors:** Hojat Anbara, Rasoul Shahrooz, Mazdak Razi, Hassan Malekinejad, Gholamreza Najafi

**Affiliations:** 1 Department of Comparative Histology & Embryology, Faculty of Veterinary Medicine, University of Tehran, Tehran, Iran; 2 Department of Comparative Histology & Embryology, Faculty of Veterinary Medicine, Urmia University, Urmia, Iran; 3 Department of Pharmacology & Toxicology, Faculty of Pharmacy, Urmia University of Medical Sciences, Urmia, Iran; 4 Department of Anatomy & Embryology, Faculty of Veterinary Medicine, Urmia University, Urmia, Iran

**Keywords:** Hemolytic anemia, Histology, Mice, Testis, Vitamin C

## Abstract

**Objective(s):**

The aim of the present study was to assess the effects of vitamin C (Vit C) on hemolytic anemia induced by phenylhydrazine (PHZ).

**Materials and Methods:**

Twenty-four healthy male mice were divided into four groups, randomly: Control group (0.1 ml/day, normal slaine, IP), PHZ group that received only PHZ 8 mg/100 g/48 hr, IP, PHZ+Vit C group that received PHZ 8 mg/100 g/48 hr, IP and Vit C 100 mg/kg BW-1/day by gavage and Vit C group that received 100 mg/kg BW-1/day Vit C by gavage. After 35 days, germinal cells, RNA damage, sperm parameters, testis malondialdehyde (MDA) content, serum total antioxidant capacity (TAC), pre-implantation embryo development and mRNA levels of cyclin D1 and c-myc in two-cell, and morula and blastocyst stages were assessed.

**Results:**

Vit C reduced the RNA damage, enhanced sperm quality, promoted pre-implantation embryo development and improved testicular antioxidant and endocrine status (*P*<0.05). Vit C reduced cyclin D1 expression and up-regulated c-myc mRNA level in two-cell, morula, and blastocyst embryonic cells.

**Conclusion:**

Vit C enhanced the fertilizing potential by ameliorating the endocrine status, antioxidant capacity, and sperm quality. Finally, the cyclin D1 and c-myc gene expressions were regulated in PHZ+Vit C treated group that promoted the embryo development.

## Introduction

Anemia is a common disorder, and several kinds of anemia have been recognized so far. It almost always results in a severe reduction in circulating red blood cells and hemoglobin (1). In case of intra or extravascular red blood cell (RBC) destruction, hemolytic anemia is a form of inherited and/or acquired anemia (2). 

Phenylhydrazine (PHZ), a member of hydrazines, has been used as a therapeutic chemical in the treatment of polycythemia Vera. According to the suggested mechanism of action of PHZ, the interaction of PHZ with hemoglobin generates hydrogen peroxide and destroys the pigment through the formation of oxidized derivatives and free radicals of hydrazine (3). Moreover, PHZ has been reported to induce generation of reactive oxygen species (ROS), which in turn results in an intensive lipid peroxidation and oxidative degradation of spectrin in the membrane skeleton (4). These characteristics of PHZ have drawn the attention of the researchers to consider PHZ as an effective chemical for studying the hemolytic anemia in experimental models (5). Vitamin C (Vit C), as a potential antioxidant agent, is able to ameliorate the oxidative-stress-related testicular impairments in animal tissues (6-10). The exogenous antioxidant therapy has also been demonstrated to improve the sperm quality in smokers and infertile patients (11). Ascorbic acid has been shown to increase the availability of the vitamins in ameliorating the oxidant-related damages. As a result of these interactions, ascorbic acid is able to fairly restore the activity of antioxidant enzymes, enable normal germ cell differentiation and up-regulate the sperm morphology and even volume (12). Likewise, Vit C has been reported to protect the sperm DNA content against exogenous oxidative stress, *in-vitro *(13). Moreover, it has been shown that the administration of Vit C in pesticide-fed animal cases improved the affected sperm morphology and significantly enhanced the sperm count (14). It has also been reported that the Vit C pretreatment prevented the hydrogen peroxide-induced sperm DNA damage (15). It has been shown that there is a direct correlation between Vit C and fertility in men (16). All mentioned antioxidant properties of Vit C may attribute to its potential ability in scavenging free radicals as well as its activating and/or regenerating properties on other endogenous antioxidants (17). Additionally, Vit C protects low density lipoproteins from oxidation and reduces the harmful oxidants in cells (18).

To the best of our knowledge, there are only a few studies regarding the effect of anemia on spermatogenesis. Moreover, the effect of antioxidant agents/chemicals against possible anemia-induced infertility is not widely investigated. Thus, the current study was designed to investigate the experimental anemia-induced histological and molecular derange-ments and to assess the protective effect of Vit C as an important water-soluble antioxidant and enzyme co-factor against anemia-induced derangements. Moreover, the pre-implantation embryo development and the roles of *Cyclin *D1 and c-myc in pre-implantation embryo development were investigated in both anemia-only and Vit C-treated animals. 

## Materials and Methods

***Animals and study design***

Twenty- four male mice (NMRI strain) weighing approximately 25-30 g were divided into four experimental groups (n = 10), randomly: Control group (0.1 ml/day, normal saline, IP), PHZ group (anemia-induced) that received only PHZ 8 mg/100 g/48 hr, IP, PHZ+Vit C-treated group that received PHZ 8 mg/100 g/48 hr, IP and Vit C 100 mg/kg BW^-1^/day by gavage, and Vit C group that received Vit C 100 mg/kg BW^-1^/day by gavage. The PHZ was purchased from Sigma Aldrich (Cas No: P6926). Vit C was purchased from Sigma Aldrich (Cas No: 8031-67-2). The acridine-orange was purchased from Sigma chemical Co. (St. Louis, MO, USA). The HTF (SAGE, ART 1020) and potassium simplex optimization medium (KSOM, Merck, MR-121-D) was provided by Elin-Teb Co. (Urmia, Iran). Two weeks before and during the entire experiments, the animals were housed in individual plastic cages with an ambient temperature of 23±3^º^C, stable air humidity, and a natural day/night cycle. The mice had free access to standard rodent laboratory, food and tap water. The procedures were carried out based on the guidelines of the Ethics Committee of the International Association for the Study of pain (19). The Urmia University research council approved this experiment.

***Necropsy and tissue sampling***

 The average body weight according to the difference between the first and last day of the treatment period (35 days) were measured. Then, the animals were euthanized with a CO_2_ gas device (Urom Adaco, Iran), and their testes were dissected out. Next, for each animal, the average weight of the testis and proportion of total body to testis weight were measured. A portion of each testis was fixed in a Bouin’s fixative solution for histological study and the rest of the samples were stored at -70 ^o^C for further studies. 

***Histological assessments***

 The fixed testes were taken out for paraffin section (5-6 µm in thickness) preparation by rotary microtome (Microm, GMBH, Germany), and stained with Hematoxylin-Eosin for histomorphometric evaluation. Histomorphometric studies were performed, and the seminiferous tubules with more than 3 germinal layers were considered as tubules with positive tubular differentiation index (TDI). The tubules with developing spermatozoa were considered as tubules with positive spermiogenesis index (SPI). The tubules with high percentages of spermatogonia type B (with dark nucleus) relative to an undifferentiated spermatogonia type A (with light nucleus) were considered as tubules with positive repopulation index (RI). The percentages of the tubules with positive TDI, SPI and RI were evaluated in 10 cross-sections from the testis of each animal in each group and the results were compared between the groups. The thickness of germinal epithelium and the diameter of seminiferous tubules were evaluated by a digital camera (Dino-Eye-AM-7023) then analyzed with Dino Capture 2.0 software for morphometric analysis.

***Fluorescent analyses for RNA damage***

The RNA damage was evaluated based on Darzynkiewicz’s method (20). In this method, the testes were washed out with ethyl alcohol and the sections were prepared using cryostat (8 µm). Then, the sections were fixed by increasing concentrations of ethyl alcohol (60%, 70%, 90% and absolute) for 15 min. Next, the sections were briefly rinsed in acetic acid (1%), followed by washing in distilled water, several times. Subsequently, the slides were stained with acridine-orange for 3 min and distained in phosphate buffer. After that, the slides were prepared to fluorescent color differentiation in calcium chloride (for 3 min). The germinal cells with damaged RNA were characterized in faint red-stained RNA. The normal cells were represented with bright red RNA in the nucleolus. 

**Table 1:** Alterations in total body weight (T.B.W), left testis weight (L.T.W)/total body weight, right testis weight (R.T.W)/total body weight and serum level of testosterone in different groups. All data are presented in Mean±SE.

**Parameters**	**Control**	**Vit C **	**PHZ**	**PHZ+Vit C**
T.B.W (g)	34.58±0.41^a^	34.66±0.44^a^	27.91±0.62^b^	31.75±0.40^a^
L.T.W/T.B.W (g)	0.0031±0.47^-4 a^	0.0031±0.87^-4 a^	0.0027±0.45^-4 b^	0.0029±0.42^-4 c^
R.T.W/T.B.W (g)	0.0036±0.56^-4 a^	0.0035±0.42^-4 a^	0.0030±0.42^-4 b^	0.0035±0.57^-4 c^
Testestrone (ng/ml)	6.49±0.18^a^	7.84±0.20^b^	4.75±0.14^c^	5.84±0.37^d^

Vit C: Vitamin C, PHZ: Phenylhydrazine. Different superscripts (a, b, c) are presenting significant differences (*P*<0.05) between the groups in the same row.

**Table 2:** Mean percentages of tubular differentiation index (TDI), repopulation index (RI), spermiogenesis index (SPI), and germinal epithelial height (G.E.H) as well as tubular diameter (T.D) in different groups. All data are presented in Mean±SE.

**Parameters**	**Control**	**Vit C **	**PHZ**	**PHZ+Vit C**
TDI (%)	88.25±2.74^a^	89.50±1.55^a^	59.21±2.73^b^	86.75±2.05^c^
RI(%)	91.50±1.22^a^	91.92±1.59^a^	63.25±2.25^b^	85.62±2.45^c^
SPI (%)	87.72±1.95^a^	85.52±1.36^a^	57.04±1.17^b^	74.66±2.83^c^
G.E.H (µm)	47.57±0.65^a^	47.96±0.89^a^	27.39±0.85^b^	33.71±0.84^c^
T.D (µm)	200.59±3.23^a^	201.03±4.32^a^	138.35±4.14^b^	174.62±5.18^c^

Vit C: Vitamin C, PHZ: Phenylhydrazine. Different superscripts (a, b, c) are presenting significant differences (*P*<0.05) between the groups in the same row.

**Table 3:** Mean average for IVF outcome in different groups, all data are presented in Mean±SE.

**Parameters**	**Control**	**Vit C **	**PHZ**	**PHZ+Vit C**
Fertilization (%)	92.26±1.27^a^	92.34±1.30^a^	58.97±0.40^b^	64.39±2.27^c^
Two-cell (%)	92.68±1.31^a^	92.51±0.71^a^	56.89±1.63^b^	63.13±2.72^c^
Four-cell (%)	85.12±2.11^a^	82.90±2.55^a^	59.82±2.68^b^	67.94±2.43^c^
Morula (%)	84.72±0.63^a^	81.53±0.68^a^	59.16±0.83^b^	65.78±2.63^c^
Blastocyst (%)	77.83±2.16^a^	77.30±1.63^a^	43.64±0.79^b^	65.08±2.24^c^

Vit C: Vitamin C, PHZ: Phenylhydrazine. Different superscripts (a, b, c) are presenting significant differences (*P*<0.05) between the groups in the same row.

**Figure 1:**
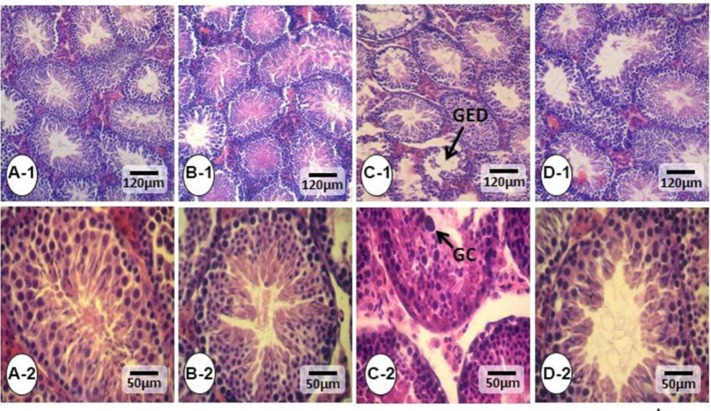
Cross section from testes: (A-1, 2) Control, (B-1, 2) Vit C, (C-1, 2) PHZ group, and (D-1,2) PHZ+Vit C groups. The intact seminiferous tubules are normal in the Control and Vit C groups (the highest magnification in section A-2). However, the seminiferous tubules from PHZ groups shows severe germinal epithelium disarrangement (arrow and the highest magnification in in section. C-2) Which shows the tubules with depleted epithelium and negative TDI and SPI. The tubules show normal, and the magnified section showed improved TDI and SPI indices. H&E staining, 400× and 800×. GED= Germinal epithelium disarrangement, GC= Giant cell

**Figure 2:**
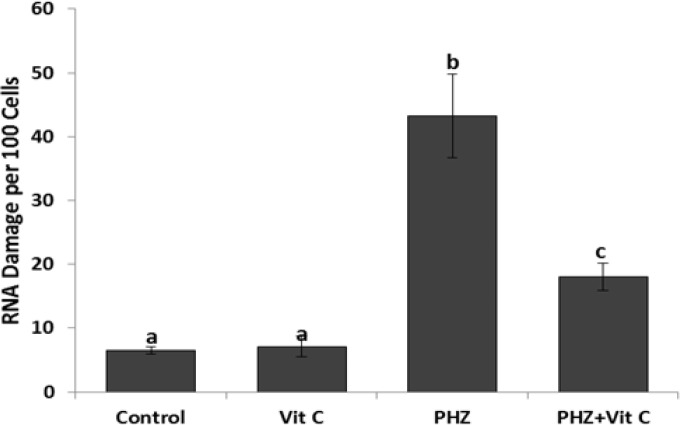
Evaluation of RNA damage: All data are presented in Mean±SE. Different superscripts (a,b,c) shows the significant differences between the different groups (*P*<0.05).

**Figure 3:**
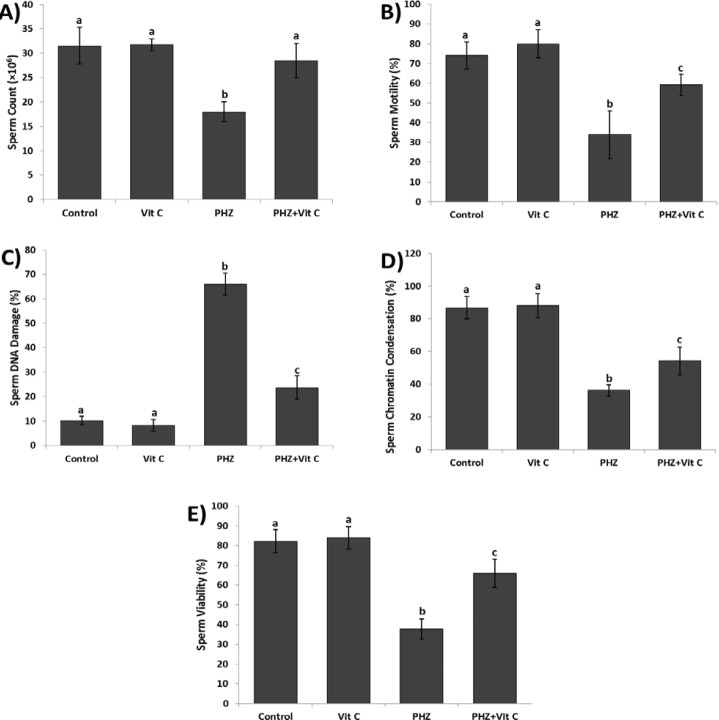
Results of sperm parameters: (**A**) Count, (**B**) Motility, (**C**) DNA damage, (**D**) Chromatin condensation and (**E**) Viability. All data are presented in Mean±SE. Different superscripts (a,b,c) shows the significant differences between the different groups (*P*<0.05).

**Figure 4:**
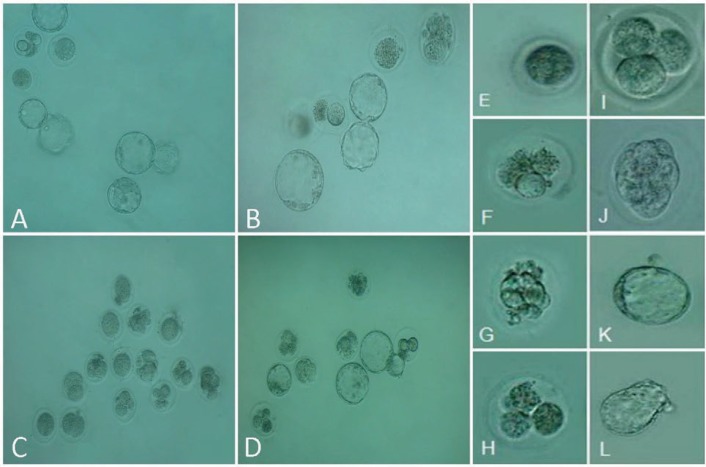
Phase-contrast microscopic view of preimplantation embryos: (A) Control, (B) Vit C, (C) PHZ and (D) PHZ+Vit C groups. (E) Unfertilized oocyte, (F) Arrest Type I, (G) Arrest Type II, (H) Arrest type III, (I) normal four-cell embryo, (J) Intact morula, (K) Intact blastocyst, and (L) Hatching stage embryo.

**Figure 5:**
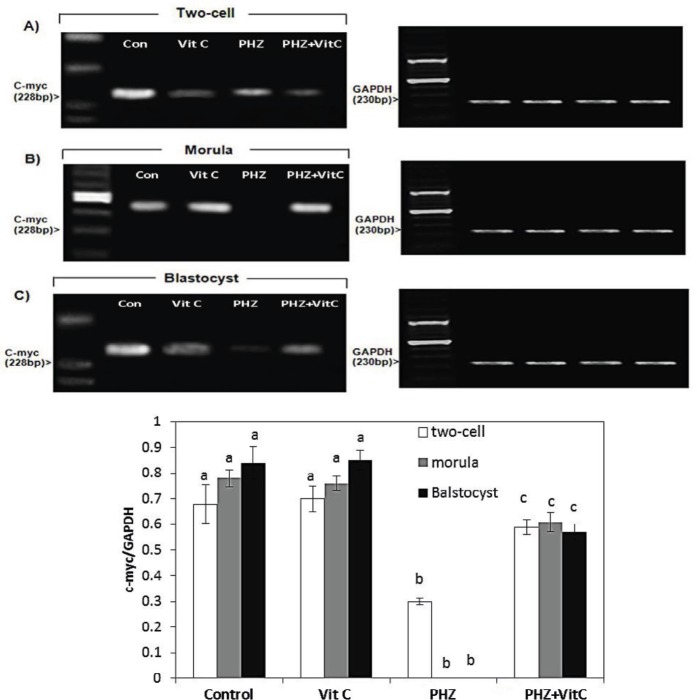
mRNA levels of c-myc in (A) Two-cell embryo, (B) Morula and (C) Blastocyst in different test and Control groups. Anemia down-regulated the mRNA level of c-myc at the two-cell embryo stage. No mRNA was exhibited at morula stage and the mRNA level was decreased at blastocyst stage. The enhanced mRNA level of c-myc in Vit C-treated, PHZ animals morula and blastocyst can be observed in this Figure. The c-myc/GAPDH density was measured by densitometry and normalized to GAPDH mRNA expression level and compared between the groups (**D**) All data are presented in Mean±SE. Different superscripts (a,b,c) shows the significant differences between the different groups (*P*<0.05).

**Figure 6:**
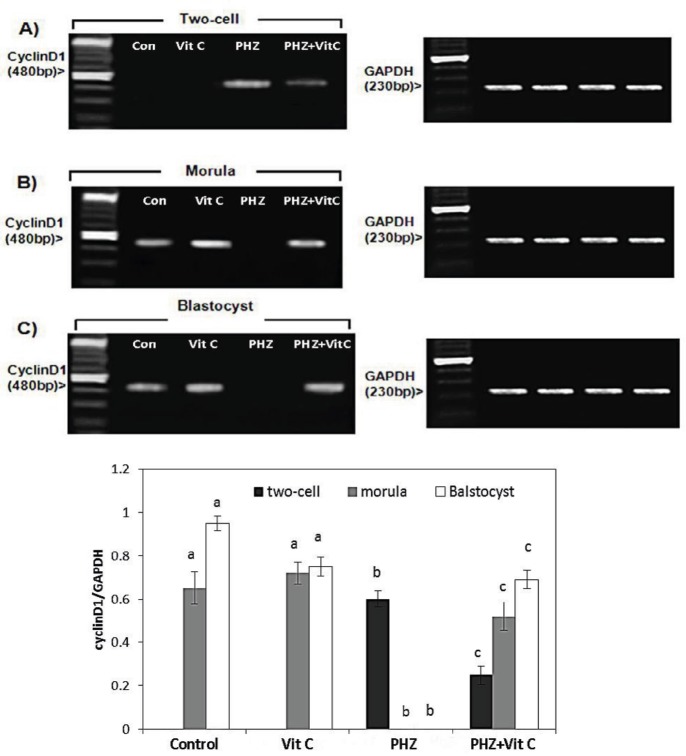
mRNA levels of cyclin D1 in (A) The two-cell embryo, (B) Morula, and (C) Blastocyst in different test and the Control groups. The cyclin D1/GAPDH density was measured by densitometry and normalized to GAPDH mRNA expression level and compared between the groups, (**D**). All data are presented in Mean±SE. Different superscripts (a,b,c) shows the significant differences between the different groups (*P*<0.05).

**Figure 7:**
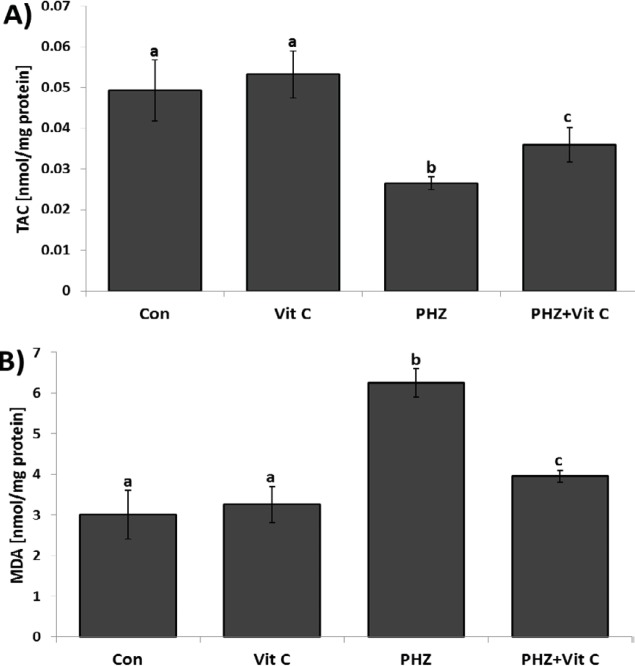
Mean TAC level (A), and MDA content (B), in the Control and other experimental groups. All data are presented in Mean±SE. Different superscripts (a,b,c) shows the significant differences between the different groups (*P*<0.05).

**Figure 8:**
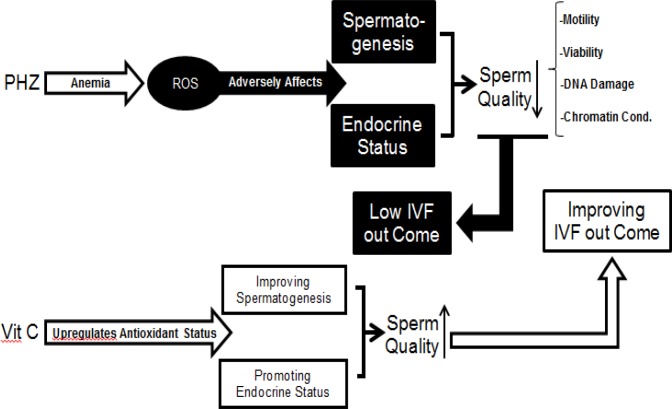
VIT C significantly ameliorated the anemia-reduced IVF outcome. Anemia resulted in oxidative stress, which in turn adversely affected the spermatogenesis and reduced the testicular endocrine status. The last evidences led to significant reduction in sperm quality. Ultimately, it was able to pathologically affect the IVF potential. However, Vit C by ameliorating the anemia-induced oxidative stress improved the spermatogenesis and provoked the endocrine status. Therefore, the increased sperm quality promoted the sperm IVF potential.

**Figure 9:**
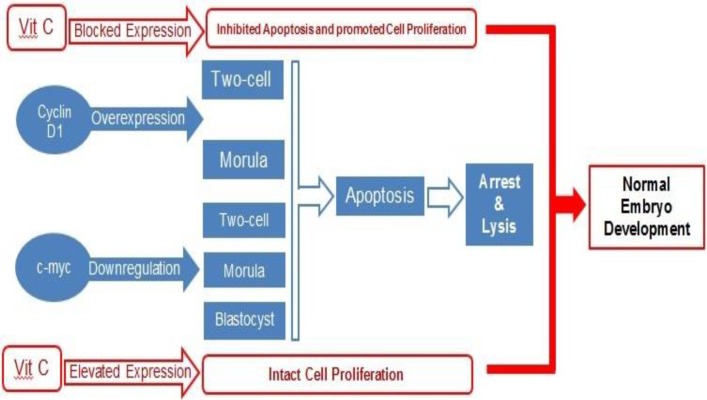
Alteration at cyclin D1 and c-myc mRNA levels. Overexpression of the cyclin D1 at two-cell and morula stages and the down-regulation of the c-myc at two-cell, morula and blastocyst stages resulted in apoptosis and embryo arrest. Vit C by a- preventing the cyclin D1 expression at two cell and morula stages and b- by ameliorating the anemia-decreased c-myc expression improved preimplantation embryos development.

***Sperm preparation and DNA damage assessment***

Epididymis was carefully refined from their surrounding tissues under a 10× magnification provided by a Stereo Zoom Microscope (TL2, Olympus Co., Tokyo). The caudate part of the epididymis was trimmed and minced in a 5 ml TCM199 medium for 30 min at 6% CO_2_, and 36.5 ^o^C in the CO_2 _equipped incubator (LEEC Co., England). After centrifugation, the sperm pellet was re-suspended in 0.5 ml of TCM199 medium. Total sperm count was performed. A small aliquot (20 µl) of sperm suspension was glass-smeared. The slides were air-dried and then fixed overnight in Carnoy’s solution (methanol/acetic acid, 3: 1) (21). Next, the slides were stained for 5 min with a freshly-prepared acridine-orange stain (AO). After washing and drying, the slides were examined using a fluorescent microscope (Leitz, Germany; excitation of 450–490 nm). On each slide, an average of 100 spermatozoa was analyzed. The percentage of spermatozoa with single-stranded DNA was calculated from the ratio of spermatozoa with red, orange, or yellow fluorescence to the total spermatozoa counted per sample.

***Sperm motility, chromatin condensation and viability***

Motility of the sperm was examined according to the WHO (WHO, 1999) standard method for manual examination of sperm motility. Accordingly, the sperm samples were diluted 1:8 in TCM199 before the examination. A 20 μl sample of the sperm was placed on a sperm examination area and examined under 10× magnification. Only the motile sperms with forward progression were counted within 10 boxes and recorded. Finally, motility was evaluated based on the following equation:

Motility(%)=[motile sperm/(motile+non-motile sperm)]×100

The eosin-nigrosin staining method was performed to assess the sperm viability (22). In brief, 50 μl of sperm was mixed with 20 μl of eosin in a sterile test tube. After 5 sec, 50 μl of nigrosin was added and mixed thoroughly. Then, the mixture of the stained sperms was smeared on the slide and examined under a bright field microscope (1,000× magnification, Olympus, Germany). The colorless sperms were considered as live and the yellow to pink stained sperms were marked as dead. The sperm count was performed according to the standard hemocytometric method as previously described by Pant and Srivastava (23). 

In addition, the aniline-blue staining was performed to analyze chromatin condensation or sperm nuclear maturity. The sperms with dark stained nuclei were detected as mature and the sperms with light blue stained nuclei were marked as immature. The sperm viability, motility, and maturity were reported in percentage and compared between groups.

***In-vitro fertilization (IVF)***

For *in-vitro* fertilization (IVF), 3 mice (from each group) were considered. In this method for inducing the superovulation, 10 IU pregnant mare serum gonadotropin (PMSG sigma, G4877) was injected intraperitoneally into each of the 6-7 week-old female mice. After 48 hr, 10 IU human chorionic gonadotrophin (HCG Sigma, C1063) was injected intraperitoneally. After 12-14 hr of HCG injection, female mice were euthanized by cervical dislocation and their oviducts were removed and the ampulla was dissected into a plastic dish containing HTF+ 4 mg/kg BSA medium. Cumulus masses surrounding the oocytes were separated and introduced into the HTF+ 4 mg/kg BSA medium. Microdrops of fertile sperm (1×10^6^ sperm /ml) in HTF+ 4 mg/kg BSA were prepared, and 10 to 15 oocytes were placed into each sperm micro drop (150 µl). The fertilization process was performed by incubation about four to six hr at 37°C under 5% CO_2._ Then, the nude and fertilized oocytes were transferred to fresh drops of HTF+ 4 mg/kg BSA medium for culture of embryos. All of the medium droplets were covered with mineral oil (sigma M8410), and fertilized oocytes were evaluated by the appearance of the pronuclei under an invert microscope with the magnification of 200**×**. Then, the zygotes were washed 3 times with KSOM and then transferred into fresh KSOM, and cultured for an additional 5 days of incubation at 37°C under 5% CO_2_. After 24 hr, the two-cell embryos rate as well as *in-vitro* embryo development were evaluated, and blastocyst stage was evaluated on the 3^rd^ day. 

***Isolation of the RNA and semi-quantitative RT-PCR***

 Eighty zygotes (two-cell), 80-90 morula, and 80-90 blastocysts were collected for reverse transcription-PCR analyses. Total RNA was isolated using Trizol reagent according to the manufacturer’s instructions (Sigma), and RT-PCR was performed using a Perkin-Elmer Gene Amp RNA PCR kit and RNA PCR Core kit (Perkin-Elmer; Norwalk, Conn). 3 µg of total RNA from each sample was used for first strand cDNA synthesis. Primers used for RT-PCR of *cyclin* D1 and *c-myc* were provided by Sina Clone Company (Karaj, Iran). The cyclin D1 primers were 5′-CGCCTTCCGTTTCTTACTTCA-3′ (Sense) and 5′- AACTTCTCGGCAGTCAGGGGA-3′ (Anti-Sense) and the product was 250 bp. C-*myc* primers were 5’-GGTTTGCTGTGGCCTCGGGATGGA-3’ (Sense) and 5′-TTCTCAGCCGCTGCCAAGCTGGTC-3′ (Anti-Sense) and the product was 228 bp. Mouse GAPDH primers were 5’-ATCTTCCAGGAGCGAGACCCCA-3’ (forward) and 5’-TCCACAATGCCAAAGTTGTCATGG-3’ (reverse), and RT-PCR product was 230 bp. The PCR conditions for *cyclin* D1 were 94˚C for 1 min, 60˚C for 1 min and 72˚C for 2 min for 40 cycles, for c-*myc* were 40 sec at 94°C, 40 sec at 65°C, and 60 sec at 72°C for 30 cycles and for GAPDH were 94˚C for 45 sec, 55˚C for 45 sec and 72˚C for 2 min for 35 cycles. RT-PCR-amplified fragments were analyzed by 2% agarose gel electrophoresis and visualized by ethidium bromide staining. Gels were scanned using an ATP Gel Documentation System and the images were analyzed using ATP software (ATPtec, Tehran, Iran) to quantify the signal intensity. The resulting bands were quantified, and the relative amount of *cyclin* D1, *c-myc* mRNAs was estimated after normalization with the GAPDH detected in the same sample.

***Assessment of malondialdehyde and total antioxidant capacity***

0.3-0.4 g of the testicular tissue was homogenized in ice-cold KCl (150 mM) and then the mixture was centrifuged at 3000 x* g* for 10 min. The supernatants were used for evaluating malondialdehyde (MDA) and 2 ml from the serum was used to evaluate the serum level of total antioxidant capacity (TAC). The assessment of TAC was carried out based on ferric reduction, antioxidant power (FRAP) assay as previously reported (24). To determine the lipid peroxidation rate, MDA content of the collected testis samples was measured using the thiobarbituric acid (TBA) reaction, as described previously (25). The amount of MDA was expressed as nmol per mg protein of the samples. The protein content of the samples was measured using the Lowry method (26).

***Evaluating serum level of testosterone***

Following 35 days, blood samples were obtained directly from the heart under light anesthesia (provided using diethyl ether). After 15 min at room temperature, the samples were centrifuged at 3000×g for 10 min to obtain the serum. The testosterone level was assessed using competitive chemiluminescent immunoassay kit (DRG Co, Germany).

***Statistical analysis***

Statistical analyses were performed using SPSS (Version 16.00, USA). The Mann-Whitney test was used for comparison of mRNA relative quantities between all groups. Finally, quantitative histomorphometric, biochemical and *in-vitro* fertilization data were compared between all groups and analyzed using one-way ANOVA, followed by Bonferroni *post-hoc* test. A *P*-value <0.05 was considered significant. All data were presented in Mean±SE.

## Results

***General findings***

Observations revealed a significant (*P*<0.05) reduction in total body weight in the PHZ-only group in comparison with the control and the PHZ+Vit C groups (*P*<0.05). The testicular weight relative to the total body weight was remarkably (*P*<0.05) decreased in PHZ-only group versus other groups. The assessment of serum testosterone in the PHZ-only group showed a significant (*P*<0.05) reduction in comparison with the control animals. Meanwhile, the PHZ+Vti C group exhibited a significant (*P*<0.05) elevation of serum testosterone (Table 1). 

***Vit C ameliorates the PHZ-induced impacts on the testis tissue***

The histological studies represented that the PHZ induced significant tubular atrophy and edema in the interstitial tissue. Nevertheless, co-administration of Vit C with PHZ inhibited the PHZ-induced derangements and reduced the tubular atrophy as well as edema. No histopathological alterations were revealed in the control and the Vit C-only groups (Figure 1). Moreover, the spermatogenesis indices were studied, and histomorpho-metric analyses represented ameliorated tubular TDI, SPI and RI indices in the PHZ+Vit C group versus the PHZ-only group (Table 2). Accordingly, the animals in the PHZ+Vit C group exhibited up-regulated percentages of tubules with positive TDI, SPI and RI indices compared to the non-treated PHZ-only group. 

***Vit C ameliorates the PHZ-induced RNA damage***

To determine the toxic effect of PHZ on the RNA contents of the cells and to estimate the protective effect of Vit C, the special fluorescent staining was performed (Figure 2). Observations revealed an intensive mRNA damage in the PHZ-only group, which was remarkably ameliorated/inhibited in Vit C co-treated animals. Accordingly, the animals in the PHZ+Vit C group exhibited a decreased number of cells/ total 100 cells (with mRNA damage) versus the PHZ-only group. Moreover, the same results were obtained using software analyses, representing a remarkable reduction in the distribution of the cells with mRNA damage/500 µm^2^ of tissue section in the PHZ+Vit C group. 

***Vit C improved the sperm parameters***

The PHZ significantly (*P*<0.05) reduced the sperm count, and diminished sperm motility and viability. Meanwhile, the PHZ+Vit C group exhibited an improved sperm count, motility and viability (*P*<0.05). More analyses for nuclear maturation and DNA integrity were conducted, and the results revealed that the anemia induced by PHZ considerably alleviated the sperm DNA damage and reduced the percentage of the sperm with chromatin condensation (*P*<0.05). In contrast, Vit C reduced the DNA damage and elevated the percentage of the sperm with chromatin condensation in the PHZ+Vit C group (Figure 3). 

***Vit C ameliorated the in-vitro fertilizing potential***

The animals in the PHZ-only group exhibited diminished fertilizing potential versus the control and the Vit C-only groups (*P*<0.05). However, co-administration of Vit C in the PHZ+Vit C group significantly increased the fertilizing potential compared to the PHZ-only group. Accordingly, the Vit C-treated animals exhibited a higher number of embryos in two-cell, four-cell, morula and blastocysts stages in comparison with the PHZ-only group. Table 3 and Figure 4 represent the IVF outcomes and embryo development, respectively.

***The results of RT-PCR for cyclin D1 and c-myc***

The RT-PCR analyses exhibited a significant reduction in the mRNA levels of *c-myc* at the two-cell zygotes and morula stages, in the PHZ-only group (*P*<0.05). However, the *c-myc* mRNA level was remarkably increased at the two-cell zygote and morula stages in the PHZ+Vit C group (*P*<0.05). Comparing the Vit C and the control groups for *c-myc* mRNA expression demonstrates no significant difference (Figure 5). Moreover, the* cyclin* D1 mRNA expression level, as an early regulator of cell cycle during the pre-implantation stage, was estimated by RT-PCR analyses. The results demonstrated that the *cyclin* D1 mRNA level was increased in two-cell embryos of PHZ-only group versus PHZ+Vit C animals (*P*<0.05). Moreover, the *cyclin* D1 mRNA expression was not determined in the control and the Vit C groups. No mRNA of *cyclin* D1 was demonstrated at morula/blastocyst stages in the PHZ-only group (Figure 6). 

***Vit C ameliorated the oxidative stress***

To assess the antioxidant status of the testicles in different groups, the tissue TAC levels were analyzed. Observations revealed a remarkable (*P*<0.05) reduction in tissue TAC level in the PHZ-only group versus the control and the Vit C-only groups. In contrast, the animals in the PHZ+Vit C group represented an increased TAC level compared to the PHZ-only group. Moreover, to estimate the oxidant-related lipid peroxidation ratio, the testicular MDA contents were evaluated and compared between groups. Biochemical findings showed a significant (*P*<0.05) enhancement of tissue MDA content in PHZ-only group compared to control and Vit C groups. However, Vit C decreased the testicular MDA content in the PHZ+Vit C group (*P*<0.05). No statistically significant differences were observed between the control and the Vit C-only groups (Figure 7). 

## Discussion

The spermatogenesis is a highly active process, which is able to produce more specific cells named sperm. Indeed, massive cell division, proliferation and differentiation during spermatogenesis result in considerable free radical generation. Thus, the balance between free radical generation and antioxidant capacity of tissue maintains the testicular hemostasis leading to progressive spermatocytogenesis, spermatogenesis and spermiogenesis. Therefore, it would be more logical to conclude that, due to the high susceptibility of the testicles against oxidative stress (27), any disruption in antioxidant defense system leads to an intensive tissue damage. In other words, it is hypothesized that anemia is able to fairly affect the male gonad because of low vascularization and its susceptibility against hypoxia-induced oxidative stress (28-30). 

Therefore, considering the potential oxidative effects of PHZ, in the present study, we focused on anemia-induced (induced by PHZ) histological and molecular damages as well as Vit C-induced ameliorative and/or protective effects against PHZ-related derangements. Our findings showed severe histological damages, severe atrophy of seminiferous tubules, edema, and reduced percentages of seminiferous tubules with positive spermatogenesis indices, including TDI, SPI and RI in the PHZ-only group (Anemia group). Moreover, anemia resulted in an intensive mRNA damage in germinal epithelium, diminished the sperm quality, suppressed testicular antioxidant capacity, decreased pre-implantation embryo development, and negatively altered the expression of the genes involved in cell cycle machinery related to embryo development. On the other hand, we found that Vit C is able to fairly reduce all of the PHZ-induced detrimental phenotypes, including spermatogenesis-related indices, mRNA content, antioxidant status, pre-implantation embryo development and cell cycle machinery. 

According to the previous studies, there is a positive correlation between oxidative stress and germ cell-related damages. Accordingly, high levels of free radicals in testicles negatively affect the germ cells, spermatozoa, somatic cells and testicular microenvironment by pathologically affecting the cellular DNA, RNA and protein structures (31-33). All these impairments are able to consequently suppress the testicular endocrine potential and result in spermatogenesis and spermiogenesis arrest (31, 34). To understand the subject, it should be noted that the testosterone indirectly amplifies/boosts the spermatogenesis by stimulating the physiological interactions of the Sertoli cells (35, 36). Taking together, we can hypothesize that Vit C by reducing the PHZ-induced oxidative stress could fairly maintain the testicular germ and somatic (such as Sertoli and Leydig Cells) cellularity. Thus, as a logical outcome, Vit C results in an enhanced testosterone synthesis, amplified Sertoli cell interaction and promoted spermatogenesis indices. In addition, it should not be ignored that the Vit C has an ability to promote the spermatogenesis through its capacity to maintain GSHdependent dehydroascorbate reductase activity. Indeed, this enzyme is abundant in the testes and is actively involved in the antioxidant defense system (37). The importance of Vit C and Vit E in spermatogenesis is defined by the fact that Vit C and/or Vit E deficiencies result in intensive oxidative stress in the testes and consequently disrupt spermatogenesis and suppress the testosterone production (38). However, ascorbate administration in healthy animals stimulates the sperm production and boosts the testosterone synthesis (39).

Considering that the sperm nuclear maturation (protamine replacement) process directly affects the DNA integrity, any impairment in this process results in a considerable DNA damage in oxidative condition (40, 41). On the other hand, the sperms with DNA fragmentation generate poor quality embryos and/or the embryos from these sperms are bound to arrest (42, 43). Considering improved sperm chromatin condensation, DNA integrity and pre-implantation embryo development in PHZ+Vit C group, we suggested that Vit C could fairly improve the pre-implantation embryo development via inducing protamine replacement and maintaining the DNA integrity of the sperms. Moreover, it has been illustrated that the reduction in sperm motility and/or impaired sperm morphology are negatively correlated with the compromised embryo development (44). Thus, similar to other parameters, enhanced sperm motility and improved sperm morphology in Vit C-treated animals, in turn, could fairly improve embryo development as well. 

In mammals, three types of *cyclins *D have been identified: D1, D2, and D3. All of these three types are able to regulate the G1/S transition of various cell types (45-49). The *cyclin *D1 was the first D-type cyclin found in mammals (50, 51). The high levels of *cyclin *D1 have been found in two stages of the cell cycle: Cell proliferation (52) and cell cycle arrest (53). Moreover, *cyclin *D1 protein was found to be up-regulated in apoptosis induction (54). In-line with our study, it has been shown that the overexpression of *cyclin *D1 at the early stages of the pre-implantation embryo development leads to cell cycle arrest and/or results in cellular apoptosis (55, 56). Considering high levels of *cyclin *D1 in the PHZ-only group and no *cyclin *D1 mRNA expression in the Vit C group, we concluded that the Vit C, by down-regulating the *cyclin *D1 expression, could fairly boost the pre-implantation embryo development. In contrast to the early stages, it has been illustrated that the suppressed expression of *cyclin *D1 in *cyclin *D1-null mice results in neurological as well as lobuloalveolar deficiencies (57). This finding suggests the essential role of *cyclin* D1 in tissue development at least during the embryonic stages. In-line with this fact, our RT-PCR analyses exhibited no expression of *cyclin *D1 in the PHZ-only group, which was significantly increased in the PHZ+Vit C group, suggesting the boosting effect of Vit C on late *cyclin *D1 expression in pre-implantation embryos. 

The mRNA of *c-myc* at the two-cell, the eight-cell and the morula stages has been identified previously (58, 59). Accordingly, it has been well-established that *myc*-family genes play an essential role in transcription and DNA replication (59, 60). Thus, it would be more logical to suggest that the reduced *c-myc* expression in the PHZ-only animals resulted in a probable deficiency in transcription and/or DNA replication process in pre-implantation embryos and consequently induced embryo development arrest at the two-cell, four-cell, eight-cell, and morula stages. Indeed, the *myc* gene families are expressed in a developmental stage-specific manner and they regulate the cell cycle process in association with a/the widely expressed *myc* target genes such as cyclins (58, 59, 61). Considering the key role of *c-myc* as transcriptional factor in cell proliferation/cycle and the correlation between these genes and the cyclins, we can conclude that alongside the PHZ-induced changes in *c-myc* and *cyclin *D1 expression, it affected the *c-myc* and *cyclin *D1 interactions by changing their expression in pre-implantation embryos. However, Vit C improved the IVF potential by regulating these gene expressions (note Figure 8 and 9). 

## Conclusion

Co-administration of Vit C ameliorated the PHZ-reduced endocrine status, increased the antioxidant capacity, and, by these mechanisms, enhanced the sperm quality and ultimately provoked the IVF potential. More analyses on the cell cycle regulators showed that Vit C improved the IVF outcomes by regulating the PHZ-altered expression of the *cyclin* D1 and *c-myc* at different stages of pre-implantation embryos.

## Conflict of interest

The current manuscript is a part of thesis for post-graduate degree as NO: D2-117, which was confirmed previously in Deputy Committee of Urmia University. 
